# Peronealsehnenpathologien: Von der Diagnose bis zur Behandlung

**DOI:** 10.1007/s00132-021-04116-6

**Published:** 2021-06-23

**Authors:** Madeleine Willegger, Lena Hirtler, Gilbert M. Schwarz, Rein﻿hard Windhager, Catharina Chiari

**Affiliations:** 1 Universitätsklinik für Orthopädie und Unfallchirurgie, Klinische Abteilung für Orthopädie, Medizinische Universität Wien, Währinger Gürtel 18–20, 1090 Wien, Österreich; 2 Zentrum für Anatomie und Zellbiologie, Abteilung für Anatomie, Medizinische Universität Wien, Wien, Österreich

**Keywords:** Peronealsehnenriss, Peronealsehnentendinopathie, Os-peroneum-Syndrom, Peronealsehnendislokation, Peronealsehnenluxation, Peroneal tendon tear, Peroneal tendinopathy, Painful os peroneum syndrome, Peroneal tendon dislocation, Peroneal tendon luxation

## Abstract

Peronealsehnenpathologien sind selten, jedoch häufig unterdiagnostiziert. Eine Assoziation mit einer chronisch lateralen Sprunggelenkinstabilität sowie mit einer varischen Rückfußachse kann bestehen. Pathologien der Sehnen lassen sich in 3 Kategorien einteilen: Tendinitis und Tenosynovitis, Sehnenrisse und Rupturen sowie Sehnensubluxation und Sehnenluxation. Die Magnetresonanztomographie ist die Standardmethode zur radiologischen Beurteilung. Die Diagnose und Behandlung basieren jedoch in erster Linie auf Anamnese und klinischer Untersuchung. Eine primär konservative Therapie kann versucht werden, ausgenommen bei Peronealsehnenluxationen des professionellen Sportlers. Die chirurgische Therapie sollte gezielt auf die zugrunde liegende Pathologie abgestimmt werden und kann dementsprechend divers von der tendoskopischen Synovektomie bis zur anatomischen Reparatur des superioren peronealen Retinakulums mit Vertiefung der retromalleolären Rinne ausfallen. Die postoperativen Ergebnisse zeigen eine hohe Patientenzufriedenheit und niedrige Reluxationsraten.

## Abstract (en)

Peroneal tendon pathologies are rare but often underdiagnosed. There may be an association with chronic lateral ankle instability as well as with varus hindfoot alignment. Pathologies of the tendons fall into three categories: tendinitis and tenosynovitis, tendon ruptures and tears, and tendon subluxation and dislocation. Magnetic resonance imaging is the standard method for radiological assessment; however, the diagnosis and treatment are based primarily on patient history and clinical examination. A primary conservative treatment can be attempted, except for peroneal tendon dislocations in professional athletes. Surgical treatment should be targeted to the underlying pathology and can accordingly vary from tendoscopic synovectomy to anatomic repair of the superior peroneal retinaculum with deepening of the retromalleolar groove. Postoperative results show high patient satisfaction and low reluxation rates.

## Lernziele

Nach Lektüre dieses Beitrags …

- ist Ihnen bekannt, welche klinischen Untersuchungen und Merkmale den Verdacht auf eine Peronealsehnenpathologie lenken,
- sind Sie mit dem anatomischen Verlauf sowie der Funktion der Peronealsehnen vertraut,
- kennen Sie die Begleitpathologien, die bei Peronealsehnenläsionen häufig vorkommen,
- sind Ihnen die bildgebenden Verfahren zur weiterführenden Abklärung geläufig,
- wissen Sie, welche Peronealsehnenpathologien am häufigsten vorkommen,
- können Sie auf Basis der rezenten Literatur Behandlungsstrategien (konservativ vs. operativ) empfehlen.

## Einleitung

Peronealsehnenpathologien und Verletzungen werden relativ gesehen selten, aber häufig unterdiagnostiziert und können eine Ursache für laterale Knöchelschmerzen sein. Ebenfalls besteht eine Assoziation mit akuten oder repetitiven Inversionstraumen des Sprunggelenks. Die **3 Hauptkategorien**

3 Hauptkategorien

der Peronealsehnenschädigungen umfassen die Tendinitis und Tenosynovitis, die Subluxation und Luxation/Dislokation sowie Sehnenrisse und -rupturen. Typischerweise sprechen diese Pathologien auf nichtoperative Behandlungen (physikalische Therapie, Verwendung von nichtsteroidalen entzündungshemmenden Medikamenten und Immobilisierung) an, in schweren oder refraktären Fällen kann jedoch ein operativer Eingriff erforderlich sein. Es wurden viele operative Verfahren zur Behandlung der Peronealsehnen beschrieben. Leider besteht die Literatur größtenteils aus retrospektiven Serien und Fallberichten, ohne dass Level-I- oder -II-Studien zur Unterstützung von Behandlungsempfehlungen verfügbar sind. Dieser Beitrag bietet einen Überblick der Epidemiologie, relevanten Anatomie, diagnostischen Aufarbeitung, biomechanischen Überlegungen, Verletzungsmechanismen und Optionen zur Behandlung von Peronealsehnenpathologien.

## Epidemiologie

Peronealsehnenpathologien sind eine seltene und unterschätzte Ursache für **laterale Rückfußschmerzen**

laterale Rückfußschmerzen

und **Funktionsstörungen**

Funktionsstörungen,

die oft übersehen werden, da es schwierig sein kann, sie von lateralen Knöchelbandverletzungen zu unterscheiden [1, 2]. In einer Studie von Dombek et al. wurden nur 60 % der Peronealsehnenerkrankungen bei der ersten klinischen Begutachtung diagnostiziert bzw. suspiziert [3]. Unbehandelt können peroneale Sehnenpathologien zu anhaltenden posterolateralen Knöchelschmerzen und erheblichen funktionellen Beschwerden führen [4, 5]. Eine Peronealsehnentendinitis und -tenosynovitis resultiert normalerweise aus verlängerter oder sich wiederholender sportlicher Aktivität, insbesondere nach einer Periode der relativen Inaktivität [1, 2]. Pathologien der Peronealsehnen verursachen häufig **chronische Knöchelschmerzen**

chronische Knöchelschmerzen

bei Sportlern (v. a. Läufern und Balletttänzern) [6], die in bis zu 77 % mit einer **chronisch lateralen Instabilität**

chronisch laterale Instabilität

einhergehen [7]. Von operativ behandelten Patienten weisen bis zu 33 % auch eine laterale Sprunggelenkinstabilität auf, die eine primäre Bandrekonstruktion erfordert. Bis zu 20 % haben eine dokumentierte Peronealsehnensubluxation, 10 % eine Insuffizienz der retromalleolären Rinne, 33 % einen tief liegenden Muskelbauch des M. peroneus brevis (PB) [2]. Peronealsehnenrisse wurden des Weiteren auch im Zusammenhang mit Fuß- und Knöchelfrakturen und mit Gicht berichtet [8, 9].

### Merke

Peronealsehnenrisse und -rupturen sind häufig assoziiert mit anderen Erkrankungen wie chronischer Tenosynovitis, schweren Inversionstraumen, Frakturen oder chronischer lateraler Instabilität des Sprunggelenks.

Isolierte Peronealsehnenrisse oder -rupturen sind selten, treten aber meist nach Inversionsverletzungen des Sprunggelenks auf [10, 11]. Die Prävalenz von PB-Längsrissen in einer humanen anatomischen Präparatstudie lag zwischen 11 % und 37 %, während Längsrisse der Sehne des M. peroneus longus (PL) seltener auftraten [12, 13]. Risse beider Peronealsehnen wurden bei bis zu 38 % der Patienten berichtet, die wegen Peronealsehnenrissen operativ behandelt wurden [14]. Dombek et al. [3] fanden einen Riss der PB-Sehne bei 88 % und einen Riss der PL-Sehne bei 13 % operativ behandelter Patienten.

Das **Alignement**

Alignement

des Sprunggelenks und des Rückfußes ist ein wichtiger Faktor, denn eine **Cavovarus-Fehlstellung**

Cavovarus-Fehlstellung

kann zu einer Überlastung der Peronealsehnen führen und für eine Tendinopathie prädisponieren. Insbesondere die PL-Sehne kann dabei betroffen sein [15].

### Cave

Eine Cavovarus-Fehlstellung des Rückfußes kann zu Überlastungen der Peronealsehnen führen.

Untersuchungen zeigten, dass zwischen 32 % und 82 % der betroffenen Patienten eine Cavovarus-Fehlstellung aufweisen [14, 15]. Peronealsehnenimpingement zwischen der Fibulaspitze und dem lateralen Kalkaneus wird auch bei Fersenvalgus (typischerweise sekundär bei Sehneninsuffizienz des M. tibialis posterior) und posttraumatischen Fehlstellungen nach Kalkaneusfrakturen mit verbreiterter Ferse beobachtet [16].

Die Luxation der Peronealsehnen ist eine relativ seltene Pathologie, die erstmals 1803 bei einem Balletttänzer beschrieben wurde und häufig mit sportlicher Aktivität in Verbindung gebracht wird, insbesondere dem Skifahren [17, 18]. **Peronealsehnenluxationen**

Peronealsehnenluxationen

können sowohl akut (traumatisch) als auch chronisch auftreten und spielen ebenfalls eine Rolle bei der chronisch lateralen Instabilität. Akute Peronealsehnenluxationen werden in bis zu 40 % der Fälle nicht diagnostiziert und oft fälschlicherweise mit einem Supinations‑/Inversionstrauma des Knöchels verwechselt [3]. Angeborene prädisponierende Faktoren für eine chronische Luxation inkludieren eine generelle Laxizität des superioren Retinaculum mm. peroneorum (SPR) oder eine flache bzw. konvexe retromalleoläre Rinne. Aufgrund der häufigen Luxationen kann es zu sekundären Längsrupturen der Sehnen kommen [19, 20].

## Anatomie

Die **Peronealmuskeln**

Peronealmuskeln

befinden sich im lateralen Kompartiment des Unterschenkels und werden durch den superfiziellen Ast des N. peroneus innerviert. Der PL entspringt dem lateralen Kondylus der Tibia und dem Fibulaköpfchen, und der PB entspringt dem mittleren Drittel der Fibula und dem intermuskulären Septum. Beide **Peronealsehnen**

Peronealsehnen

treten etwa 4 cm proximal der Spitze des lateralen Malleolus in eine gemeinsame Synovialscheide ein. Sie verlaufen posterior zum lateralen Malleolus durch einen fibroossären Tunnel, die sog. **retromalleoläre Rinne**

retromalleoläre Rinne

(„groove“) oder auch retromalleolärer Sulcus genannt, wobei die PL-Sehne posterolateral zur PB-Sehne liegt. Die PB-Sehne sitzt anterior des PL, wenn die Muskeln in den retromalleolären Tunnel eintreten, und ist auf dieser Höhe flach oder schalenförmig um die PL-Sehne angeordnet. Beide Sehnen verlaufen um die distale Fibula herum, passieren oberflächlich das Lig. calcaneofibulare und treten in separate Tunnel entlang des anterioren Fortsatzes des Kalkaneus ein. Diese Tunnel verlaufen parallel und werden durch das Tuberculum peronei geteilt, wobei der PB dorsal zum Tuberculum und der PL plantar verläuft [21, 22]. Die retromalleoläre Rinne wird posterolateral durch das **superiore peroneale Retinaculum**

superiore peroneale Retinaculum

(SPR), anterior durch die Fibula und medial durch die hinteren talofibulären, kalkaneofibulären und posterior-inferioren tibiofibulären Bänder gebildet. Die Rinne ist mit Faserknorpel ausgekleidet und variiert anatomisch in Breite und Tiefe.

**Figure 1:**
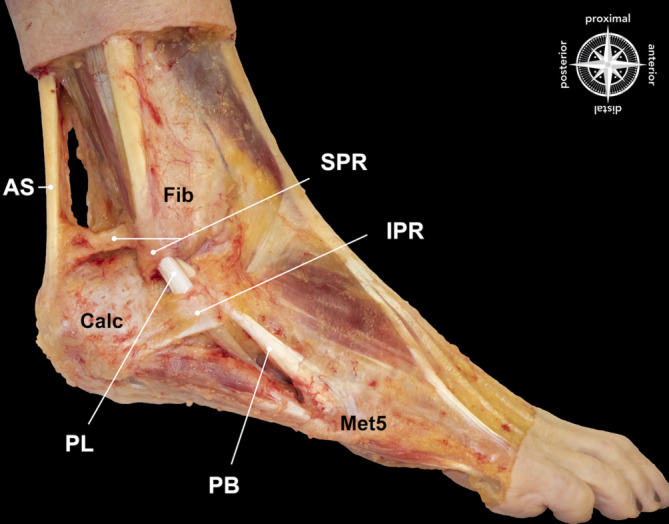

Das SPR fungiert als primärer Stabilisator der Peronealsehnen auf Höhe des Außenknöchels, welches die Subluxation bzw. Luxation der Sehnen aus der Rinne verhindert. Es handelt sich um ein etwa 1–2 cm breites fibröses Band, das von der posterolateralen Seite des distalen Teils der Fibula ausgeht und eine variable Insertion aufweist. Es wurden 5 verschiedene Insertionsvariationen beschrieben. Der häufigste Typ mit ca. 47 % umfasst 2 Bänder: ein oberes Band, das am anterioren Aspekt der Achillessehnenscheide ansetzt, und ein unteres Band, das am lateralen Aspekt des Kalkaneus am Tuberculum peronei inseriert ([23]; Abb. 1).

Distal zum Knöchel gabelt sich die Sehnenscheide um das Tuberculum peronei, während die Peronealsehnen den lateralen Aspekt des Kalkaneus kreuzen. Die Peronealsehnen verlaufen durch das IPR ca. 2–3 cm distal zur Fibulaspitze. Die **PB-Sehne**

PB-Sehne

führt direkt zu ihrer Insertion an der Basis des fünften Mittelfußknochens [24]. Die **PL-Sehne**

PL-Sehne

verläuft medial zwischen der Cuboid-Rinne und dem Lig. plantare longum und inseriert an der plantaren Oberfläche der Basis des ersten Mittelfußknochens und des medialen Keilbeins. Das **Os peroneum**

Os peroneum

ist ein oval bis rundes Ossikel, das sich in der Substanz der PL-Sehne auf Höhe des Calcaneocuboidgelenks befindet. Sarrafian [22] beschreibt, dass das Os peroneum vorwiegend fibrokartilaginär angelegt ist, aber bei ca. 20 % der Personen verknöchert und bei nur 5 % röntgenologisch sichtbar ist. Seine Morphologie variiert, und ähnlich wie bei anderen Sesambeinen im Körper kann es ein-, zwei- oder mehrteilig sein. Das Os peroneum kann mit dem lateralen Kalkaneus, mit dem Calcaneocuboidgelenk oder dem inferioren Anteil des Cuboids artikulieren.

### Merke

Das Os peroneum ist ein Sesambein in der Sehne des M. peroneus longus.

Als akzessorisches Ossikel kann ein sog. Os vesalianum an der Basis des Metatarsale 5 im Bereich der Insertion der M. peroneus brevis Sehne auftreten. Meist wird es als radiologischer Zufallsbefund diagnostiziert, in seltenen Fällen kann es jedoch zu Schmerzen des lateralen Mittelfußes führen [25].

Die Peronealsehnen erhalten ihre **Blutversorgung**

Blutversorgung

über Vinculae aus der posterioren Peronealarterie und der A. tarsalis medialis. Diese Vinculae durchdringen den posterolateralen Aspekt jeder Sehne über den gesamten Verlauf in der retromalleolären Rinne [26]. Es wird in der Literatur diskutiert, dass die Peronealsehnen kritische **avaskuläre Zonen**

avaskuläre Zonen

aufweisen, die potenziell zur Entwicklung einer Tendinopathie beitragen können. Petersen et al. [27] beschrieben 3 avaskuläre Zonen: eine in der PB-Sehne auf Höhe der Wende um den lateralen Malleolus und 2 in der PL-Sehne. Die erste avaskuläre Zone des PL ist ebenfalls bei der Wende um den Außenknöchel, die zweite tritt dort auf, wo sich die Sehne um das Os cuboideum wendet. Diese avaskulären Zonen entsprechen auch den häufigsten Lokalisationen der Peronealsehnentendinopathien. Das Vorhandensein avaskulärer Zonen wurde jedoch von mehreren Autoren widerlegt, sodass das Mikrogefäßsystem der Peronealsehnen weiterhin umstritten ist [26].

Es wird vermutet, dass mehrere anatomische Variationen zur Entstehung von Peronealsehnenpathologien beitragen. Der **retromalleoläre Sulcus**

retromalleoläre Sulcus

variiert in Größe und Form, was die Stabilität der Peronealsehnen beim Gleiten hinter der Fibula beeinträchtigen kann. Die Untersuchung von 178 Fibulae in einer humanen anatomischen Studie ergab, dass 82 % einen konkaven retromalleolären Sulcus hatten, 11 % waren flach, und 7 % hatten eine konvexe Oberfläche [28]. Der Sulcus hat einen Durchmesser von 6–7 mm und ist 2–4 mm tief. Lateral wird er durch einen fibrokartilaginären Kamm verstärkt. Die Form der Rinne wird durch diesen Knorpelkamm und nicht durch die Konkavität der Fibula selbst bestimmt [29]. Eine flache oder schmale retromalleoläre Rinne kann zu einer Sehnensubluxation und nachfolgenden Peronealsehnenläsion beitragen. Die Beschaffenheit des knorpeligen Kamms und des SPR erscheinen für die biomechanische Stabilität der Sehnen wichtiger zu sein als die tatsächliche Geometrie des Sulcus [30, 31]. Ein tief liegender PB-Muskelbauch ist relativ häufig und wird bei bis zu 33 % gesunder Personen beobachtet. Per Definition spricht man von einem **tiefen Muskelbauch**

tiefen Muskelbauch

sobald er sich unter den oberen Rand des SPR erstreckt. Klar ist auch, dass die Position des Muskelbauchs abhängig ist von der Sehnenexkursion. Der pathologische Wert dieser anatomischen Variation ist fragwürdig. Innerhalb der retromalleolären Rinne wurden 2 akzessorische Muskeln beschrieben: der **M. peroneus quartus**

M. peroneus quartus

und der **M. peroneus quintus**

M. peroneus quintus

mit einer Inzidenz von 10–22 % bzw. 18–34 %. Beide Muskeln können aus dem PB, dem PL, der Fibula, dem M. peroneus tertius oder einer Kombination dieser Strukturen entspringen. Ihre Insertionen unterscheiden sich jedoch. Der M. peroneus quartus fügt sich normalerweise mit einem Sehnenzug dem M. extensor digitorum longus an oder setzt entlang des peronealen Tuberkels des Kalkaneus an, während der M. peroneus quintus typischerweise am dorsalen Aspekt des fünften Mittelfußknochens inseriert [32]. Diese zusätzliche/n Sehne/n kann/können zu einer Enge im retromalleolären Sulcus führen und dadurch Schmerzen auslösen. Eine MRT(Magnetresonanztomographie)-Studie detektierte als assoziierte Pathologien Längsrisse der PB-Sehne und eine mögliche Subluxation oder Dislokation der Peronealsehnen.

Orthopädische Chirurgen und Radiologen sollten sich der Existenz dieser Muskeln bewusst sein, nicht nur wegen der möglichen assoziierten Pathologien, sondern auch wegen seiner potenziellen Verwendung bei Rekonstruktionen des SPR oder bei Tenodesen [33].

### Cave

Der M. peroneus quartus und M. peroneus quintus können mit einer zusätzlichen Sehne im retromalleolären Sulcus verlaufen.

Die Hypertrophie des Tuberculum peronei wurde auch als Ursache von Peronealsehnenerkrankungen beschrieben. Diese anatomische Variation erhöht die mechanische Belastung der Peronealsehnen, was möglicherweise zu einer Tendinopathie und einer Einschränkung des normalen Gleitens innerhalb der Sehnenscheiden führt [34].

## Funktion der Peronealsehnen und biomechanische Aspekte

Der PB abduziert und evertiert den Fuß, zusätzlich fungiert er als Plantarflexor im oberen Sprunggelenk. Der PL evertiert ebenfalls den Fuß, plantar flektiert den ersten Strahl und arbeitet als sekundärer Plantarflexor im oberen Sprunggelenk. Während der Standphase des Gangzyklus stabilisiert er zusätzlich die mediale Säule des Längsgewölbes. Die Peronealmuskeln sind Antagonisten des M. tibialis posterior, M. flexor hallucis longus, M. flexor digitorum longus und M. tibialis anterior. Zusammen machen sie 63 % der gesamten **Rückfußeversionskraft**

Rückfußeversionskraft

aus, wobei der M. peroneus longus 35 % und der M. peroneus brevis 28 % ausmachen. Die peroneale Muskelgruppe übt nur 4 % der gesamten Plantarflexionskraft auf das Sprunggelenk aus, was verglichen mit dem M. triceps surae (M. gastrocnemius und M. soleus) (87 %) relativ schwach ist. Die Peronealsehnen spielen ebenso eine wichtige Rolle als dynamische Stabilisatoren des lateralen und medialen Knöchelbandkomplexes [2]. Bei einem plötzlichen Inversionstrauma kontrahiert sich die Peronealgruppe als Erstes. Eine verzögerte Aktivierung der Peronealmuskeln wird dementsprechend als Ursache einer funktionellen Instabilität des oberen Sprunggelenks gewertet. Die Studienlage zu diesem Thema ist jedoch widersprüchlich [35, 36].

## Klinische Untersuchung

Vor der klinischen Untersuchung sollte eine gründliche **Anamnese**

Anamnese

durchgeführt werden. Eine detaillierte Anamnese sollte das Vorhandensein von assoziierten Erkrankungen beinhalten, wie beispielsweise rheumatoide Arthritis, Psoriasis, Hyperparathyreoidismus, diabetische Neuropathie, Fersenbeinfrakturen, die Einnahme von Fluorchinolonen und lokale Steroidinjektionen erhöhen die Prävalenz von Peronealsehnenfehlfunktionen [2]. Bevor sich der Untersucher auf die laterale Seite des Sprunggelenks konzentriert, empfiehlt es sich, die Aufmerksamkeit auf die Gesamtausrichtung des Beins und das Alignement des Rückfußes zu richten. Patienten mit einem **Rückfußvarus**

Rückfußvarus

haben ein erhöhtes Risiko für Überlastungen der Peronealmuskulatur bzw. der Peronealsehnen. Die biomechanischen Veränderungen durch die Fehlstellung des Rückfußes erhöhen einerseits die resultierenden Kräfte auf die Peronealgruppe, prädisponieren jedoch auch zu Verletzungen im Sinne von vermehrten Inversionstraumen, andererseits kann der Varus auch das Resultat einer Peronealschwäche sein. Die Flexibilität und Korrigierbarkeit des Varus sollten beurteilt werden, da dies Auswirkungen auf die Behandlung mit Orthesen haben kann. Eine Varusferse könnte den Untersucher auf eine zugrunde liegende neuromuskuläre Störung, wie z. B. Charcot-Marie-Tooth, aufmerksam machen. Es sollte auch auf eine Schwächung der intrinsischen Muskeln, Krallenbildung der Kleinzehen, oder eine Plantarflexion des ersten Strahls geachtet werden. Die Patienten können auch eine nicht-neurologische oder subtile Pes-Cavus-Ausrichtung des Rückfußes aufweisen. Zusätzlich sollte die ligamentäre Stabilität des lateralen Sprunggelenks mit der vorderen Schublade und dem **Talar-Tilt-Test**

Talar-Tilt-Test

überprüft werden. Peronealsehnenpathologien demaskieren sich oft durch eine Schwellung hinter der distalen Fibula oder am lateralen Kalkaneus, eine Empfindlichkeit bei Palpation entlang des Sehnenverlaufs, Schmerzen bei widerständiger Eversion (Verdacht auf PB), passiver Inversionsdehnung oder widerständiger Plantarflexion des ersten Strahls (Verdacht auf PL).

### Merke

Eine schmerzhaft aktive Eversion gegen Widerstand lässt eine Reizung der PB-Sehne vermuten, ein Schmerz bei Plantarflexion des 1. Strahls gegen die Hand des Untersuchers ist typisch für eine gereizte PL-Sehne.

Peronealsehnenrisse zeigen sich typischerweise mit starken posterolateralen Knöchelschmerzen und Schwellungen entlang der Sehnenscheide. Diese Symptome sind in der Regel schwerwiegender bei jüngeren Patienten, während ältere Patienten völlig asymptomatisch sein können [5, 37]. Eine aktive Zirkumduktion des Knöchels kann eine Sehnensubluxation hervorrufen oder provozieren. Sobel et al. [38] beschrieben den **peronealen Kompressionstest**

peronealen Kompressionstest,

der zur Beurteilung von Schmerzen, Krepitus und Schnappen am hinteren Rand der distalen Fibula bei kräftiger Knöcheleversion und Dorsalextension verwendet wird. Bei diesem Manöver wird manueller Druck entlang der Peronealsehnenscheide in der retromalleolären Rinne ausgeübt, wobei das Knie um 90° gebeugt ist und sich der Fuß in einer ruhenden Plantarflexionsstellung befindet [16, 38].

Die Injektion eines **Lokalanästhetikums**

Lokalanästhetikums

in die Sehnenscheide kann helfen, den Schmerz an den Peronealsehnen zu lokalisieren, in 15 % der Fälle besteht jedoch eine Kommunikation der Sehnenscheide mit dem oberen Sprunggelenk oder Subtalargelenk. Die Verwendung von Steroiden kann generell nicht befürwortet werden, da das Risiko einer späteren Sehnenruptur gegeben ist.

### Merke

Eine diagnostische Infiltration mit Lokalanästhetikum in die Sehnenscheide kann während der diagnostischen Abklärung hilfreich sein.

## Radiologische Diagnostik

Ein anteroposteriores und laterales **Röntgenbild**

Röntgenbild

des symptomatischen Knöchels und Fußes in Belastung sind hilfreich, um die Fußmorphologie, wie z. B. einen Pes cavus, zu bestimmen. Röntgenbilder der kontralateralen Seite können zum Vergleich erforderlich sein. Knochenpathologien wie Stressfrakturen, posttraumatische Deformitäten, Knochentumoren und Arthrosezeichen, die zu den Symptomen beitragen können, sollten ebenfalls durch **Nativröntgen**

Nativröntgen

ausgeschlossen bzw. erkannt werden. **Schrägaufnahmen**

Schrägaufnahmen

des Fußes können ein vergrößertes Tuberculum peronei sowie ein Os peroneum zeigen. Das Os peroneum ist meist auf der dorsoplantaren Aufnahme nicht sichtbar, da es sich genau an der Umlenkstelle der PL-Sehne unterhalb des Calcaneocuboidgelenks befindet und deshalb häufig knöchern verdeckt ist. Das „**Fleck-Zeichen**

Fleck-Zeichen

“ oder englisch „fleck sign“ auf Röntgenbildern ist pathognomonisch für eine knöcherne SPR-Avulsion und kann auf eine Sehnensubluxation oder -dislokation hinweisen. Vor allem im traumatologischen Bereich sollte bei unklarer Anamnese eines akuten Inversions‑/Supinationstraumas des Sprunggelenks auf anteroposterioren Röntgenbildern immer darauf geachtet werden.

Die **Ultraschalluntersuchung**

Ultraschalluntersuchung

bietet den Vorteil einer dynamischen Echtzeitdarstellung der Peronealsehnen und kann eine Subluxation identifizieren [39]. Grant et al. [40] berichteten in der Studie mit präoperativer Ultraschalluntersuchung über eine 90 % Genauigkeit bei der Diagnose von Peronealsehnenrissen. Der positive prädiktive Wert der Ultraschalluntersuchung zur Erkennung einer Peronealsehnensubluxation wurde sogar mit 100 % angegeben [39, 41]. Die primäre Limitation der Ultraschalluntersuchung sind natürlich die Untersucherabhängigkeit sowie eine flache Lernkurve.

**Computertomographien**

Computertomographien

sind am besten geeignet, um die knöcherne Anatomie zu beurteilen, wie beispielsweise die Morphologie des retromalleolären Sulcus, ein hypertrophiertes Tuberculum peronei oder ein Impingement der Sehnen durch eine Deformität der lateralen Wand des Kalkaneus nach Fersenbeinfraktur.

Die **Magnetresonanztomographie**

**Figure 2:**
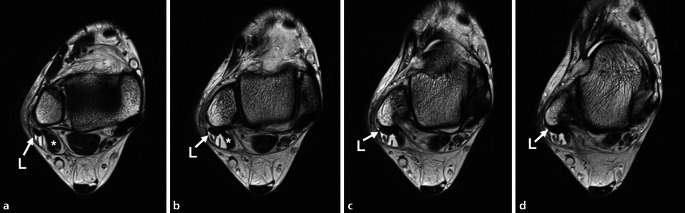

Magnetresonanztomographie

hat den Vorteil, dass eine gleichzeitige Pathologie des Sprunggelenks, die potenziell für die Symptome verantwortlich sein kann, wie z. B. eine subtalare Arthropathie, talare Knorpeldefekte oder ein Os peroneum, demaskiert werden kann. Eine Tendinose und Tenosynovitis der Peronealsehnen lassen sich am besten auf T2-gewichteten oder axialen protonendichtegewichteten Bildern darstellen. Eine erhöhte Signalintensität innerhalb der Sehne und ein Flüssigkeitssignal um die Sehne kennzeichnen eine Tendinitis (Abb. 2).

**Zirkumferenzielle Flüssigkeit**

Zirkumferenzielle Flüssigkeit

innerhalb der gemeinsamen Peronealsehnenscheide, die breiter als 3 mm ist, ist hochspezifisch für eine peroneale Tenosynovitis [42]. Ein Riss des PB kann als c‑förmige, halbierte Sehne oder als erhöhtes intratendinöses T2-Signal erscheinen [43]. Ein Riss des PL kann durch einen linearen oder runden Bereich mit erhöhtem Signal innerhalb der Sehne [44], eine flüssigkeitsgefüllte Sehnenscheide, ein Knochenmarködem entlang der lateralen Kalkaneuswand oder ein hypertrophiertes Tuberculum peronei detektiert werden [45]. In einer Studie zeigte die Magnetresonanztomographie zur Diagnose von PB-Rissen eine Sensitivität von 83 % und eine Spezifität von 75 % im Vergleich zu den intraoperativen Befunden [46]. Steel und DeOrio [47] berichteten, dass die Spezifität der Magnetresonanztomographie für die Erkennung von Rissen der PB, PL und beider Sehnen bei 80 %, 100 % und 60 % liegt. Sie stellten jedoch fest, dass die MRT-Untersuchung weniger nützlich für die Diagnose von anatomischen Anomalien wie einem tief liegenden PB Muskelbauch oder einem Peroneus-quartus-Muskel ist. Der sog **„magic angle effect“**

„magic angle effect“

muss berücksichtigt werden, bevor die Diagnose eines Peronealsehnenrisses ausgeschlossen wird. Dieser tritt auf, wenn die Sehnen einem gekrümmten Weg um den lateralen Malleolus folgen, wodurch die Sehnenfasern 55° zur magnetischen Achse stehen, was zu einem artefaktreichen Signal führt [48]. Der Effekt kann subtile Veränderungen einer Tendinose maskieren und senkt die Sensitivität und Spezifität nachweislich auf etwa 75–80 %. T2-gewichtete Bilder (oder eine beliebige Sequenz mit langer Echozeit), bei denen der Fuß in Plantarflexion gelagert wird, sind hilfreich bei der Reduzierung dieses Artefakts [49]. Brandes und Smith [15] fanden jedoch heraus, dass die Magnetresonanztomographie dazu neigt, Pathologien der Peronealsehnen zu überschätzen. Obwohl die Magnetresonanztomographie die Beurteilung von Erkrankungen der Peronealsehnen erleichtern kann, sollten die endgültige Diagnose und Behandlung in erster Linie auf der Anamnese und der körperlichen Untersuchung beruhen [14].

Die **Peronealtenographie**

Peronealtenographie

ist ein invasives und stark anwenderabhängiges bildgebendes Verfahren, das derzeit für die Diagnose von Peronealsehnenerkrankungen nur bedingt geeignet ist, da es weitgehend durch die Magnetresonanztomographie bzw. durch die Tendoskopie ersetzt wurde.

Van Dijk und Kort [50] waren 1998 die Ersten, die über die **Tendoskopie**

Tendoskopie

der Peronealsehnen berichteten. Das distale Portal wird zunächst 2 cm distal der Fibulaspitze, in einer Linie mit der Sehne, platziert. Unter Verwendung einer 2,7 mm 30°-Optik und einer Kochsalzlösungsinsufflation der Sehnenscheide wird dann das proximale Portal 2 cm proximal der Fibulaspitze angelegt. In erster Linie handelt es sich um ein diagnostisches Instrument, es kann aber für eine einfache Tenosynovektomie und die Lösung von Adhäsionen verwendet werden [51].

## Tendinitis, Tenosynovitis, Tendinose

Patienten mit einer Reizung bzw. Überlastung der Peronealsehnen stellen sich typischerweise mit posterolateralen Knöchelschmerzen vor, die sich bei Aktivität verschlimmern und bei Ruhe bessern. Normalerweise besteht eine Empfindlichkeit über den Peronealsehnen, wobei eine tastbare Raumforderung, die sich mit der Sehne bewegt, auf eine Tendinose hindeutet. Der Zustand ist durch Verdickung, fokale Sehnendegeneration und Schwellung gekennzeichnet und tritt häufiger im inframalleolären Anteil auf. Häufig sind knotige Verdickungen, Risse oder Einrisse der Sehne vorhanden [16].

Die Peronealsehnentendinopathie sollte zunächst konservativ behandelt werden. **Konservative Maßnahmen**

Konservative Maßnahmen

bestehen aus nichtsteroidalen entzündungshemmenden Medikamenten, Ruhe, Eis, Kompression und Einschränkung der Aktivität. **Physikalische Therapie**

Physikalische Therapie,

die Dehnungs‑, Kräftigungs- und propriozeptive Übungen umfasst, kann hilfreich sein. Die Behandlung mit einer Einlage basiert primär auf dem Alignement des Fußes bzw. Rückfußes, das mit dem Coleman-Block-Test ermittelt wird. Ein lateraler Fersenkeil kann bei primären Varusdeformitäten des Rückfußes hilfreich sein, während ein laterales Vorfußpolster vorteilhafter ist, wenn der Vorfußvalgus die primäre Deformität ist. Bei therapierefraktären Fällen kann die Ruhigstellung in einer starren Knöchel-Fuß-Orthese, einem CAM(„controlled ankle motion“)-Stiefel oder einem Gehgips für 6 Wochen versucht werden [2].

### Cave

Häufige Kortisoninjektionen sollten aufgrund der Gefahr einer konsekutiven Sehnenruptur vermieden werden.

Wenn die konservative Behandlung versagt, besteht die Operation typischerweise aus einer **offenen Synovektomie**

offenen Synovektomie.

Die Sehnenscheide wird in Längsrichtung eröffnet, und jeder degenerierte Sehnenbereich wird debridiert. Assoziierte Varianten oder Pathologien wie ein M. peroneus quartus oder ein hypertrophiertes Tuberculum peronei sollten entsprechend behandelt werden (Exzision des Muskels bzw. Glättung/Abtragung des knöchernen Vorsprungs) [52]. Die Sehnenscheide wird offen belassen, um eine postoperative Stenosierung zu verhindern. Postoperativ wird das Sprunggelenk in einem Unterschenkelgips in leichter Eversion und Plantarflexion entlastend für 2 Wochen mobilisiert, gefolgt von einer weiteren 2‑ bis 4‑wöchigen Ruhigstellung in Neutralstellung unter Belastung. Bewegungsumfangs- und Kräftigungsübungen können ab der 4. bis 6. Woche postoperativ begonnen werden [53].

## „Painful os peroneum syndrome“

**Figure 3:**
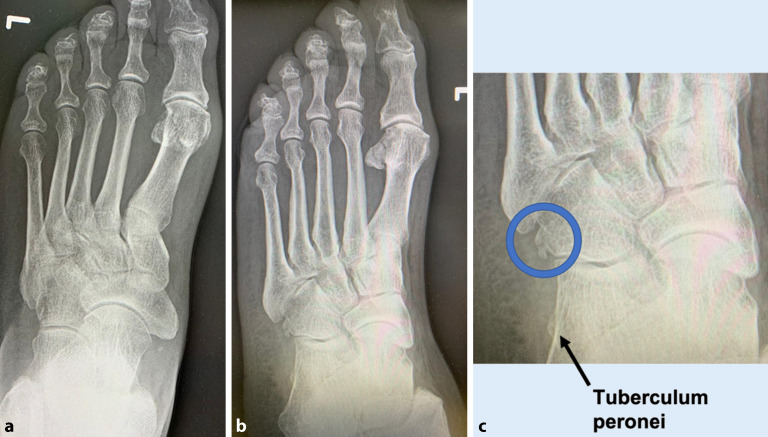

Das schmerzhafte Os-peroneum-Syndrom (POPS) ist ein von Sobel et al. [54] geprägter Begriff, der ein Spektrum von **posttraumatischen Zuständen**

posttraumatischen Zuständen

der Peronealsehnen beschreibt. Das Syndrom umfasst eine akute Fraktur des Os peroneum oder eine Diastase eines mehrteiligen Os peroneum, eine chronische Fraktur des Os peroneum in Verbindung mit einer stenosierenden Tenosynovitis des PL, eine partielle oder vollständige Ruptur der PL-Sehne in der Nähe des Os peroneum oder eine Einklemmung der PL-Sehne und des Os peroneum durch ein hypertrophiertes Tuberculum peronei (Abb. 3).

Die konservative Behandlung des POPS ähnelt derjenigen der Tendinitis und umfasst die **Ruhigstellung**

Ruhigstellung

in einem belastenden Gipsverband und möglicherweise Kortikosteroidinjektionen. Bei therapierefraktären Schmerzen kann die Indikation zur **Operation**

Operation

gestellt werden. Die PL-Sehne wird auf Höhe der „cuboid notch“ freigelegt, und eine Markierungsnaht wird in den distalen Anteil gelegt. Das Os peroneum wird aus der Sehne herausgeschält, und wenn ein Defekt verbleibt, wird eine direkte Reparatur durchgeführt. Ist eine Reparatur nicht möglich, sollte proximal eine Tenodese des PL an die PB-Sehne durchgeführt werden, wobei der degenerierte Abschnitt des PL exzidiert wird [16]. Derzeit gibt es nur Evidenz der Stufen IV und V für die Behandlung des POPS [55].

## Peronealsehnenrisse und -rupturen

Die Ätiologie von Peronealsehnenrupturen bleibt umstritten. Munk und Davis [56] schlugen 2 mögliche pathogene Mechanismen für Rissläsionen der PB-Sehne vor. Ein Mechanismus besteht darin, dass eine Subluxation der PB-Sehne als Folge einer Laxizität oder eines Risses des SPR durch chronische Sprunggelenkinstabilitäten oder Inversionsverletzungen auftritt. Wenn die Sehne subluxiert, kann die PB-Sehne über die scharfe posterolaterale Kante der Fibula spreizen oder spalten. Bei dieser Theorie folgt die Spaltläsion der Subluxation. Der zweite potenzielle Mechanismus beschreibt eine Kompression der PB-Sehne zwischen der PL-Sehne und der posterioren Fibula. Während eines Inversionstraumas kommt es zu einem Längsriss der Sehne. Bei diesem vorgeschlagenen Mechanismus folgt die Subluxation des lateralen Anteils der PB-Sehne der Spaltläsion. Der zweite Mechanismus erklärt, warum PB-Sehnenrisse in Abwesenheit einer Sehnensubluxation gefunden werden. Andere anatomische Faktoren, die ebenfalls zu Peronealsehnenrissen beitragen können, werden im Abschnitt „Epidemiologie“ ausführlich besprochen.

PL-Sehnenrisse können akut oder chronisch sein. **Akute Risse**

Akute Risse

der PL-Sehne sind in der Regel die Folge einer Sportverletzung oder eines Traumas [57]. Diese Verletzungen inkludieren einen Riss der Sehne, die Avulsion der Sehne am oder durch das Os peroneum oder Dislokation der Sehne über den lateralen Malleolus. Die hohen Scherspannungen innerhalb der Sehne, während sie sich unter dem Fuß dreht, können ein wesentlicher Faktor sein, der zur Pathomechanik von Längsrissen beiträgt. Weder das Vorhandensein eines Os peroneum scheint die Sehne für einen Riss zu prädisponieren, noch ist das Os peroneum an den meisten Rissen beteiligt. Jeder Zustand, der zu einer Überbeanspruchung des PL führt (z. B. Cavovarus-Deformität, chronisch laterale Instabilität), kann eine **chronische Verletzung**

chronische Verletzung

hervorrufen [53].

Die Behandlung der Peronealsehnenrisse kann primär konservativ erfolgen, tatsächliche Rupturen sind meist Domäne der chirurgischen Therapie. Ursprünglich als anatomische Klassifikation für PL-Risse beschrieben, ist die **Brandes-Smith-Klassifikation**

**Figure 4:**
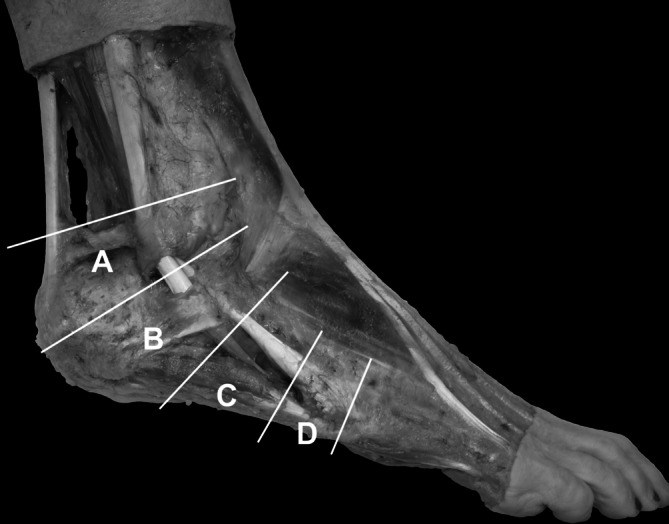

Brandes-Smith-Klassifikation

sowohl für PB- als auch PL-Risse nützlich ([15]; Abb. 4).

**Sehnenlängsrisse**

Sehnenlängsrisse

sollten primär mit einem **Débridement**

Débridement,

gefolgt von einer **Tubularisierungstechnik**

**Figure 5:**
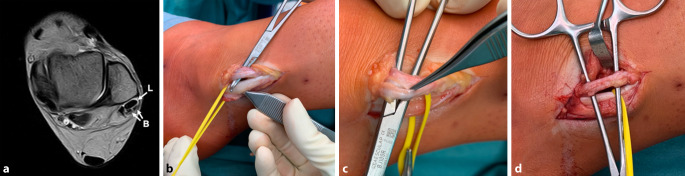

Tubularisierungstechnik

repariert werden. Über einen lateralen Zugang wird die retromalleoläre Rinne durch Inzision des SPR eröffnet, wobei eine Manschette auf dem fibrokartilaginären Kamm für eine evtl. Reparatur und einen Verschluss belassen wird. Die Oberfläche jeder Sehne wird inspiziert. Nach Débridement des Sehnenlängsrisses und Exzision der ausgedünnten Anteile wird die Sehne tubularisiert, sofern mehr als 50 % der PB-Sehne verbleiben. Dies entspricht einer Grad-1-Läsion nach Krause und Brodsky [5]. Diese Technik zielt darauf ab, die ausgedünnte Sehne wieder tubulär (röhrenförmig) zu formen. Dabei wird eine laufende, nichtresorbierbare Naht (z. B. 3‑0) entlang der Innenfläche der Sehne gesetzt. Anschließend wird mit einer resorbierbaren Naht (z. B. 5‑0) die Sehne umwendelt, sodass die ursprüngliche Form wiederhergestellt wird (Abb. 5).

Wenn nach dem Débridement < 50 % der Sehne verbleiben, handelt es sich um eine Grad-2-Läsion, und eine **Tenodese**

Tenodese

wird durchgeführt [5].

### Merke

Verbleiben > 50 % der PB-Sehne nach Débridement erfolgt die Tubularisation.

Die Tenodese besteht aus der Exzision des degenerierten Anteils des PB und der Naht des proximalen und distalen Endes der PB-Sehne an den PL. Dazu wird eine Side-to-Side-Technik angewandt, wobei das proximale PB-Sehnenende mindestens 3 cm oberhalb des Außenknöchels und das distale Ende 5 cm unterhalb der Fibulaspitze tenotomiert wird. Dadurch werden eine mögliche Stenose und Beeinträchtigung der Reparatur in der retromalleolären Rinne und der lateralen Kalkaneuswand vermieden. Die Erfolgsraten nach einer Tenodese sind hoch und liegen bei ca. 70–80 % mit einer Rückkehr zur Aktivität nach ca. 12 Wochen. Redfern und Myerson [14] haben einen detaillierten Behandlungsalgorithmus für die intraoperative Beurteilung von Peronealsehnenrissen publiziert. In diesem Algorithmus werden auch Optionen eines Sehnentransfers sowie zweizeitige Verfahren mit Silicon-Spacer und Autograft/Allograft-Rekonstruktion bei chronischen Sehnenrupturen abgebildet (Tab. 1).

**Table 1:** 

Redfern und Myerson Typ	Pathologischer Befund	Behandlung
*I*	Beide Sehnen sind grob intakt	Exzision/Débridement des Längsrisses und Tubularisierung der verbleibenden Sehne
*II*	Eine Sehne ist gerissen und irreparabel, und die andere Sehne ist funktionsfähig (hat ausreichende Exkursion)	Tenodese proximal zwischen M.-peroneus-longus- und M.-peroneus-brevis-Sehne
*III*	Beide Sehnen sind nicht funktionsfähig	–
a	Keine Exkursion des proximalen Muskels	Sehnentransfer
b	Exkursion des proximalen Muskels und keine Vernarbung der Sehnenscheide	Einzeitige Sehnentransplantation
c	Exkursion des proximalen Muskels mit Vernarbung der Sehnenscheide	Zweizeitige Sehnentransplantation

Risse beider Sehnen können auf Steroidinjektionen, Diabetes mellitus, rheumatoide Arthritis und Verletzungen im Zusammenhang mit Subluxation, Dislokation und Instabilität der Peronealsehnen zurückgeführt werden. Wapner et al. [58] behandelten gleichzeitige Risse mit einem Hunter-Rod der distal am freien Ende der Sehne angebracht wurde, um eine Synovialscheide zu etablieren. Die zweite Operation erfolgte 3 Monate später, mit einem M.-flexor-hallucis-longus(FHL)-Transfer und Pulvertaft-Naht zur Rekonstruktion der Peronealsehnen. Bei solchen Verfahren spricht man von einer „salvage procedure“. Nach einer Nachuntersuchung von 8,5 Jahren waren 6 der 7 Patienten schmerzfrei. **Sehnentransfers**

Sehnentransfers

des FHL oder FDL (M. flexor digitorum longus) zur Adressierung von Peronealsehnenrupturen werden allerdings kontrovers diskutiert. Das Gremium der ESSKA-AFAS empfiehlt keinen Sehnentransfer, da das Verfahren mehrere biomechanische Einschränkungen aufweist und längerfristig mit erheblichen Defiziten bei Kraft und Gleichgewicht verbunden ist [59]. Die ESSKA-AFAS-Konsenserklärung [60] spricht sich in Fällen, in denen die Reparatur eines oder beider Sehnenrisse nicht möglich ist, für die Verwendung von **autologen Transplantaten**

autologen Transplantaten

(z. B. Hamstring) aus. Diese werden aufgrund ihrer mechanischen und biologischen Eigenschaften gegenüber Allograft-Transplantaten bevorzugt. Das Gremium bevorzugt eine Sehnentransplantation gegenüber einem Tenodeseverfahren, hauptsächlich weil die Tenodese das biomechanische Gleichgewicht des Fußes direkt beeinflusst. Eine biomechanische Präparatstudie von Pellegrini et al. [61] fand eine unzureichende Spannung der Peronealsehnen nach einer Tenodese des PB zum PL, während ein Allograft mit einer erheblichen Wiederherstellung der Spannung verbunden war.

## Peronealsehnensubluxation oder -luxation/-dislokation

Die Subluxation und Dislokation der Peronealsehne(n) werden oft als akute oder chronische Verletzung unterschieden. Obwohl Subluxation und Dislokation seltene Ursachen für Schmerzen im lateralen Sprunggelenk sind, können beide zu erheblichen Beeinträchtigungen führen.

### Merke

Eine Peronealsehnensubluxation beschreibt eine pathologische Sehnenposition in der retromalleolären Rinne, wobei das SPR intakt ist.

Eine **Subluxation**

**Figure 6:**
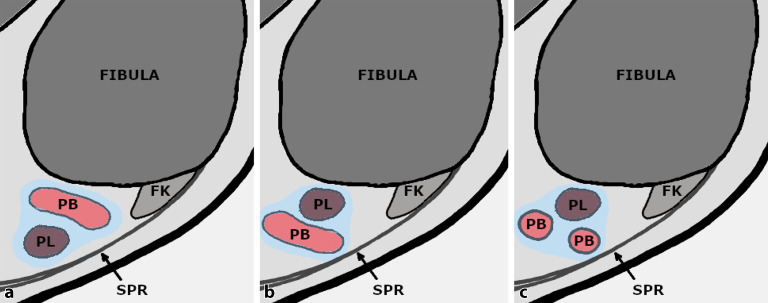

Subluxation

bezieht sich auf die Position der Sehnen innerhalb der retromalleolären Rinne, während das SPR intakt bleibt. Die Patienten präsentieren sich mit Schmerzen und tastbarem Schnappen der Sehnen bei der Zirkumduktion des Knöchels. Raikin et al. [62] definieren 2 Typen: Bei Typ A schnappen die PB- und PL-Sehne übereinander und tauschen ihre relativen Positionen (die PL-Sehne kommt tief und medial zur PB zu liegen), ohne dass es zu einem Riss in den Sehnen oder einer Unterbrechung des SPR kommt. Beim anderen Subtyp (Typ B) subluxiert die PL-Sehne durch einen longitudinalen Spaltriss innerhalb der PB-Sehne, wobei ein Teil der PL-Sehne auf dieser Höhe tief an der PB-Sehne zu liegen kommt. Das SPR ist intakt (Abb. 6).

Der erste Fall einer **Peronealsehnenluxation**

Peronealsehnenluxation

wurde 1803 von Monteggia [63] beschrieben. Sehr häufig treten sie bei Sportlern und professionellen Athleten auf, Skifahren wird als eine der typischen Sportarten genannt [64]. Eine Luxation tritt auf, wenn eine oder beide Sehnen aus der retromalleolären Rille verschoben werden, was typischerweise durch eine plötzliche exzentrische Kontraktion der Peronealmuskulatur gegen eine akute Plantarflexion des invertierten Fußes oder eine erzwungene Dorsalextension während der Eversion des Fußes hervorgerufen wird. Die PL-Sehne neigt aufgrund ihrer anatomischen Lage zwischen der PB-Sehne und dem SPR eher zur Luxation als die PB-Sehne [65]. Wie bereits erwähnt, sind v. a. eine flache bzw. konvexe retromalleoläre Rinne und eine varische Rückfußachse Risikofaktoren, die eine chronisch laterale Instabilität und Peronealsehnenpathologie begünstigen.

Im Jahr 1976 untersuchten Eckert und Davis [66] 73 Patienten mit Verletzungen des SPR und klassifizierten 3 Arten von Verletzungen. Verletzungen des Grades I (51 %) waren durch eine Avulsion des SPR vom lateralen Malleolus gekennzeichnet, wobei die Sehnen zwischen Knochen und Periost lagen. Bei Verletzungen des Grades II (33 %) wurde der fibrokartilaginäre Kamm mit dem SPR abgerissen, die Sehnen liegen zwischen fibrokartilaginärem Kamm und Fibula. Bei Verletzungen des Grades III (16 %) ist ein dünnes kortikales Knochenfragment von der Fibula avulsiert. Im Jahr 1987 fügte Oden [64] dieser Klassifizierung den Grad IV hinzu, bei dem das SPR von seinem hinteren Ansatz am Kalkaneus und der verbindenden Faszie zur Achillessehne gerissen ist, die Sehne gleitet durch den SPR-Defekt und liegt oberflächlich zum SPR.

Laut ESSKA-AFAS-Konsenserklärung sollte bei der Behandlung von Peronealsehnenluxationen vorrangig auf 2 Dinge Rücksicht genommen werden:

1. ob es sich um eine akute oder chronische Verletzung handelt und
2. ob es sich um einen professionellen Sportler handelt.

Für die Behandlung von Peronealsehnenluxationen wurden mehrere **Behandlungsoptionen**

**Figure 7:**
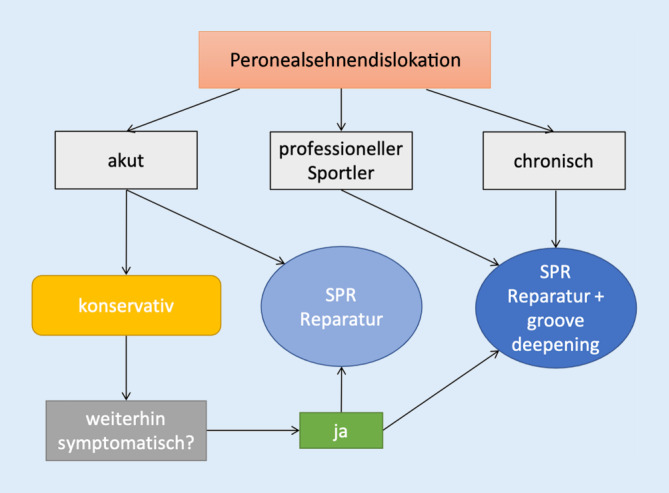

Behandlungsoptionen

vorgeschlagen, die im Allgemeinen darauf abzielen, das SPR zu reparieren oder zu rekonstruieren, prädisponierende Faktoren zu korrigieren und das Volumen des peronealen Tunnels bzw. der retromalleolären Rinne zu vergrößern. Während die Vorteile einer Operation in der Literatur aufgezeigt wurden [67], bleibt der Wert der konservativen Behandlung unklar. Die aktuelle Evidenz beschränkt sich auf eine Reihe von Fallberichten und kleinen retrospektiven Serien, die darauf hindeuten, dass das Risiko einer erneuten peronealen Instabilität bei etwa 50 % liegt [68]. Bei akuter Instabilität bei Nichtsportlern kann sowohl eine konservative als auch eine chirurgische Behandlung indiziert sein (Abb. 7).

**Figure 8:**
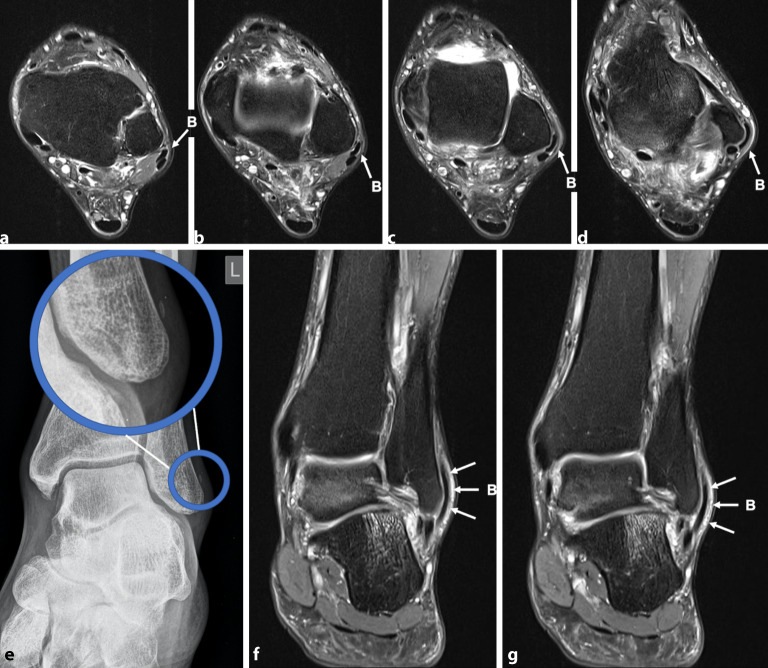

Obwohl die konservative Behandlung mit einem 50 %-Risiko des Scheiterns behaftet ist, führt die **sekundäre chirurgische Behandlung**

sekundäre chirurgische Behandlung

nicht zu einer schlechteren Prognose oder verändert die verfügbaren chirurgischen Optionen nicht. Die konservative Behandlung sollte die Ruhigstellung in einem Gips in leichter Plantarflexion oder in einem Walker-Stiefel mit einem 2 cm hohen Fersenkeil für 6 Wochen beinhalten. Wenn der Patient jedoch eine vermutete oder bestätigte Verletzung des anterioren talofibulären Ligaments hat, sollte er in einer neutralen Position immobilisiert werden, um die Heilung dieses lateralen Ligaments nicht zu gefährden. Nach 6 Wochen wird eine physikalische Therapie mit Kräftigung des Peronealgruppe und Knöchelpropriozeptionsübungen eingeleitet. Der professionelle Sportler und auch chronische Luxationen sollten operiert werden (Abb. 8).

Die Operation besteht aus der Reposition der Sehnen in die retromalleoläre Rinne und der Reparatur des SPR. Mittels direkter **SPR-Reparatur**

SPR-Reparatur

mit transossären Nähten oder mittels Fadenanker wurden ausgezeichnete klinische Ergebnisse mit rascher Genesung berichtet [17, 69]. Eine zusätzliche **Rinnenvertiefung**

Rinnenvertiefung

(„groove deepening“) kann erforderlich sein. Dies kann mittlerweile auch endoskopisch, aber auch klassisch offen erfolgen [60]. Bei der offenen Rinnenvertiefung wird das SPR inzidiert, die Peronealsehnen werden anterior disloziert, und dann wird mit einem Meißel ein knöcherner Lappen von der posterolateralen Seite der Fibula angehoben. Die darunter liegende Spongiosa wird dann mit einer Fräse bis zu einer Tiefe von ca. 5 mm entfernt. Der Lappen wird wieder reponiert und impaktiert. Die Sehnen werden wieder in die Rinne verlagert, und das SPR wird wieder repariert oder rekonstruiert [17].

**Knochenblockmethoden**

Knochenblockmethoden

wie beispielsweise die Operation nach Kelly sind aufgrund der hohen Komplikationsraten aus der Mode gekommen. Die distale Fibula wird nach einer Sagittalosteotomie entweder rotiert oder nach posterior translatiert, wodurch eine mechanische knöcherne Barriere für die Peronealsehnendislokation geschaffen wird. Die berichteten Komplikationen inkludierten die postoperative Verschiebung des Knochenkeils, Heilung in Fehlposition, Irritation und Schmerzen durch Schrauben und Sehnenabrieb [70]. Ein systematischer Review befasste sich 2015 mit dem Thema der Sportfähigkeit sowie der klinischen Ergebnisse nach chirurgischer Behandlung von Peronealsehnenluxationen. Die chirurgische Behandlung der peronealen Sehnenluxation bietet gute Ergebnisse, hohe Zufriedenheit und eine schnelle Rückkehr zum Sport. Bei Patienten, die sowohl mit Rinnenvertiefung als auch mit SPR-Reparatur behandelt wurden, sind die Raten für die Rückkehr zum Sport signifikant höher. Die **Redislokationsrate**

Redislokationsrate

beträgt bei Langzeitnachuntersuchungen weniger als 1,5 % [67].

### Merke

Die Reluxationsrate nach operativer Behandlung einer Peronealsehnenluxation liegt bei unter 1,5 %.

Wenn die Operation die Reparatur des SPR beinhaltet, sollte die Rehabilitation mit 2 Wochen ohne Belastung in einem Unterschenkelgips beginnen, gefolgt von 4 Wochen Belastung in einem Gipsverband oder einem Walker-Stiefel. Zwei Wochen nach dem Eingriff kann mit aktiven Bewegungsübungen gestartet werden. Es ist wichtig, dass die Sehnen erst 6 Wochen nach der Reparatur des SPR belastet werden [60].

## Fazit für die Praxis

- Beim posterolateralen Knöchelschmerz sowie bei akuten Inversions‑/Eversionstraumen des Sprunggelenks sollte immer auch an eine Peronealsehnenpathologie gedacht werden.
- Peronealsehnenpathologien sind oft mit einer chronisch lateralen Instabilität des Sprunggelenks sowie einer Cavovarus-Fehlstellung des Rückfußes assoziiert.
- Die Tendoskopie hat sowohl einen diagnostischen als auch therapeutischen Stellenwert.
- Konservative Therapiemaßnahmen sollten primär immer versucht werden – außer bei der Peronealsehnenluxation des professionellen Sportlers, wo eine primäre chirurgische Therapie empfohlen wird.
- Die Reparatur des SPR (superiores Retinaculum mm. peroneorum) mit oder ohne „groove deepening“ hat sich als operative Therapie der Wahl bei Peronealsehnenluxation etabliert.

## CME-Fragebogen

Welche zusätzliche Pathologie des Sprunggelenks ist am häufigsten bei einer Tenosynovitis bzw. bei Rissen der Peronealsehnen vorliegend?

Osteochondritis dissecans

Chronisch laterale Instabilität

Rückfußvalgus

Dorsales Impingement

Achillessehnenruptur

Welche akute Peronealsehnenpathologie wird häufig (bis zu 40 %) fälschlicherweise mit einem Supinations‑/Inversionstrauma des Knöchels verwechselt?

Tendinitis der Sehne des M. peroneus longus

Längsriss der Sehne des M. peroneus brevis

Ruptur der Sehne des M. peroneus longus

POPS („painful os peroneum syndrome“)

Luxation der Sehne des M. peroneus longus

Welche anatomische Struktur gilt als primärer Stabilisator der Peronealsehnen auf Höhe des Knöchels?

Superiores Retinaculum mm. peroneorum

Inferiores Retinaculum mm. peroneorum

Tuberculum peronei (Kalkaneus)

Lateraler Malleolus

Medialer Malleolus

Welche Muskelsehne verläuft bei ca. 20 % der Patienten durch den retromalleolären Sulcus?

Sehne des M. flexor digitorum brevis

Sehne des M. plantaris

Sehne des M. peroneus accessorius

Sehne des M. flexor digitorum quintus

Sehne des M. peroneus quintus

Wo ist der anatomische Ansatz der Sehne des M. peroneus brevis?

Basis des Os metatarsale 4

Basis des Os metatarsale 5

Os cuboideum

Os naviculare

Basis des Os metatarsale 1

Ein 63-jähriger Patient kommt aufgrund von länger bestehenden Knöchelschmerzen links in Ihre Fußspezialambulanz. Anamnestisch besteht kein Trauma, die Schmerzen haben vor ca. 3 Monaten nach einem Wanderwochenende begonnen. Der niedergelassene Orthopäde hat ein Röntgenbild des Sprunggelenks (mit altersentsprechendem Normalbefund) anfertigen lassen. Zusätzlich wurde mehrmals eine lokale Infiltration „hinter dem Knöchel“ durchgeführt. Bei der klinischen Untersuchung fällt Ihnen eine kraftvolle Plantarflexion des ersten Strahls gegen Widerstand auf, jedoch die Eversion und Abduktion links sind im direkten Seitenvergleich mit rechts abgeschwächt. Die periphere Sensibilität in beiden unteren Extremitäten ist seitengleich. Die Schmerzen sind dorsal der distalen Fibula links lokalisiert. Welche Peronealsehnenpathologie vermuten Sie nach Erhebung der Anamnese und der klinischen Untersuchung?

Ruptur des superioren Retinaculum mm. peroneorum

Ruptur der Sehne des M. peroneus brevis

Ruptur der Sehne des M. peroneus longus

POPS („painful os peroneum syndrome“)

Luxation der Sehne des M. peroneus longus

Bei welcher radiologischen bildgebenden Technik kann der „magic angle effect“ die Diagnose von Peronealsehnenpathologien erschweren?

Röntgen

Computertomographie

Magnetresonanztomographie

Tenographie

Tendoskopie

Ein Patient mit Schmerzen am lateralen Fußrand wird Ihnen von einem Kollegen mit der Verdachtsdiagnose „POPS“ zugewiesen. Wofür steht diese Abkürzung?

„Posterior peroneal syndrome“

„Painful operated pes syndrome“

„Painful os peroneum syndrome“

„Plantar oblique pain syndrome“

„Plantar ossification pain syndrome“

Sie haben nach Versagen der konservativen Therapie bei MRT(Magnetresonanztomographie)-verifizierter Längsruptur der Sehne des M. peroneus brevis nun die Indikation zur Operation gestellt. Intraoperativ zeigt sich nach dem Darstellen der Peronealsehnen die Längsruptur der PB(M. peroneus brevis)-Sehne, wie bereits vermutet. Die PL(M. peroneus longus)-Sehne ist intakt. Nach dem Débridement der PB-Sehne sind noch ca. 60 % des ursprünglichen Sehnendurchmessers vorhanden. Nach Krause und Brodsky handelt es sich um eine Grad-1-Läsion, dementsprechend führen Sie weiter den folgenden Operationsschritt durch:

Verschluss der Sehnenscheide nach Débridement

Tubularisation der Sehne

Tenodese der PB-Sehne auf die PL-Sehne mit Side-to-Side-Technik

Tenodese der PB-Sehne auf die PL-Sehne mit Pulvertaft-Nähten

Tenotomie und Resektion der PB-Sehne

Eine junge aufstrebende Skifahrerin hat beim Testen neuer Skischuhe während eines Probetrainings einen Sturz erlitten. Sie beschreibt eine forcierte Dorsalextension und Eversion des Fußes im Schuh. Seitdem bestehen starke Schmerzen dorsal der distalen Fibula, begleitet von einer Schwellung. Die Patientin wurde im Unfallkrankenhaus erstbegutachtet, und ein Röntgen zeigte ein „fleck sign“. Daraufhin wurde ein Unterschenkelspaltgips angelegt. Sie suspizieren eine knöcherne SPR(superiores Retinaculum mm. peroneorum)-Avulsion mit begleitender Peronealsehnenluxation. Welche Behandlung empfehlen Sie, basierend auf der aktuellen Studienlage?

Konservative Therapie mit 6 Wochen Unterschenkelgips entlastend

Konservative Therapie mit 2 Wochen Unterschenkelgips entlastend, gefolgt von 2 bis 4 Wochen Walker-Schuh

Konservative Therapie mit Unterschenkelgips in leichter Plantarflexion oder in einem Walker-Stiefel mit einem 2 cm hohen Fersenkeil für 6 Wochen

Operative Therapie mit Reposition der Sehne und Knochenblocktechnik nach Kelly

Operative Therapie mit Reparatur des SPR und ggf. Vertiefung der retromalleolären Rinne

## Abkürzungen

ESSKA-AFAS: European Society of Sports Traumatology, Knee Surgery & Arthroscopy—Ankle and Foot Associates
FDL: M. flexor digitorum longus
FHL: M. flexor hallucis longus
IPR: Inferiores Retinaculum mm. peroneorum
MRT: Magnetresonanztomographie
PB: M. peroneus brevis
PL: M. peroneus longus
PD FS: „Proton density fat saturated”
POPS: „Painful os peroneum syndrome”
SPR: Superiores Retinaculum mm. peroneorum
TSE: Turbo-Spin-Echo
